# Commentary on the United Kingdom evidence report about the effectiveness of manual therapies

**DOI:** 10.1186/1746-1340-18-4

**Published:** 2010-02-25

**Authors:** Scott Haldeman, Martin Underwood

**Affiliations:** 1 Department of Neurology, University of California, Irvine, USA; 2 Department of Epidemiology, School of Public Health, University of California, Los Angeles, USA; 3 Primary Care Research, Warwick Medical School Clinical Trials Unit, University of Warwick, UK

## Abstract

This is an accompanying commentary on the article by Gert Bronfort and colleagues about the effectiveness of manual therapy. The two commentaries were provided independently and combined into this single article by the journal editors.

## Introduction

This paper is two commentaries on the article by Gert Bronfort and colleagues about the effectiveness of manual therapy [1]. The first commentary is provided by Professor Scott Haldeman and the second by Professor Martin Underwood.

## Discussion

### Evidence informed and guided clinical practice: a clinician's point of view by Professor Scott Haldeman

Bronfort et al [1] are to be congratulated on the production of this review of the clinical studies and systematic reviews of the scientific literature that have been published on the efficacy of the manual therapies and other treatments commonly offered by chiropractors. Although there are multiple other more detailed systematic reviews on the management of specific disorders I am not aware of any publication that has addressed the broader scope of manual therapy and chiropractic. His document should be of value to all chiropractors, medical physicians who work closely with chiropractors, as well as payers and health care policy makers. Although it is possible to argue over specific wording and disagree on the quality of some of the quoted studies in this document it is not possible to question the depth and scientific integrity of this work.

Although I have been very active as a panellist or chairman of evidence based guidelines for a number of associations (the American Academy of Neurology, the North American Spine Society, the United States (US) Government Agency for Health Care Policy and Research (AHCPR), the Bone and Joint Decade 20000-2010 Task Force on Neck Pain and Its Associated Disorders (NPTF), Guidelines for Chiropractic Quality Assurance and Practice Parameters, the American Academy of Occupational and Environmental Medicine, the California Department of Industrial Relations) my primary means of making a living for the past 40 years has been the care of patients in a private clinical practice. The question that I and other clinicians raise when reviewing this type of study is: "how can I use the conclusions and information to improve the care I provide to my patients?"

I have a specific interest in guidelines of this type in that my primary practice is in the medical specialty of neurology with a special interest in spinal disorders. Most of my patients are referred for consultation and expect me to provide information on the treatment options available to them including medications, surgery, injections, rehabilitation, the different manual and chiropractic treatments and other complementary approaches to their health.

One common response to the publication of evidence based guidelines that clinicians do not fully understand, is anger that their clinical experience and observations are discounted and their common practice procedures are being questioned. When the AHCPR Guidelines were published in the US on Acute Low Back Pain and did not endorse surgery for uncomplicated low back pain due a lack of evidence there was a national outcry followed by political attacks by surgeons that led the US Congress to prohibit further government agencies from producing guidelines. The recent fury by the United Kingdom (UK) pain specialists that led to the forced resignation of the president of their society after publication of the UK NICE Guidelines that was critical of the research supporting injections for back pain is another example of the difficulty clinicians have in accepting the assessment of the efficacy of their treatment approach. I would be surprised if practicing chiropractors whose clinical observations, like those of their medical counterparts in the above situations, suggest that they are helping patients with a number of conditions where the evidence for efficacy is either non-existent or contradicts their own experience will simply accept the conclusions in this document without further discussion.

It is, however, a serious mistake to try to attack or disagree with the evidence when treating patients. It does not serve patients to provide treatment that has been shown to be ineffective or where there is insufficient evidence to reach a conclusion when there are other options available that have been demonstrated to be beneficial. It is not acceptable today to claim that a treatment is effective in helping patients when there is no evidence to support these claims. It does not help the reputation of a profession that is striving to be considered the authority in a field, if practitioners are unwilling to understand and practice according to the latest clinical evidence.

Chiropractors are extremely fortunate in these times of evidence based health care. There was a time, not long ago, when there was little or no evidence to support the practice of manipulation that is the mainstay of chiropractic practice. There were also widely advertised claims that manipulation could have very serious complications and therefore should not be offered patients in the absence of evidence. There has, however, been a rapid growth in the number of clinical trials that have studied the effectiveness of manipulation, mobilization and massage over the past 20 years and, as this document demonstrates, there is now little dispute amongst knowledgeable scientists that manipulation is of value in the management of back pain, neck pain and headaches that make up 90% or more of all patients who seek chiropractic care. At the same time, a close review of the evidence, including the recent large population studies in Ontario [2], have demonstrated that the incidence of serious side effects such as stroke following chiropractic care is extremely rare and is probably not related to manipulation in most patients but due to the fact that patients develop neck pain or headache as a result of a dissection of a vertebral artery that progresses through the natural history of dissection to stroke irrespective of the clinician the patient consults.

It is not unexpected, however, that numerous claims made by chiropractors over the years, based on their clinical observations, have not stood up to critical analysis and the results of studies often suggest that these observations are due to placebo or the natural course of the disorder rather than the actual treatment. This has been true of a vast number of medical treatments. A recent Special Issue of The Spine Journal on Evidence Informed Management of Chronic Low Back Pain listed over 200 treatments currently being offered patients with low back pain, most of which are offered by medical physicians [3]. Of these, less than 10% have a reasonable body of support based on high quality clinical trials. The greatest research support was for therapies commonly used by chiropractors including the manual therapies, education and exercise.

My goal as a clinician is to ensure that I offer the highest quality of care to patients based on the best available knowledge. I find that this is easy to do and patients greatly appreciate, and in fact expect, care that has research support. In my personal practice I incorporate evidence such as that noted in this report in the following manner when caring for my patients:

1. Ensure that I attend the scientific meetings where the latest clinical studies are presented and discussed.

2. Ensure that I keep up to date with the latest research in order to be confident that I am as knowledgeable about my field of practice as any other clinician.

3. Ensure that when I advertise my practice or talk to prospective patients that I only make claims that I can support by quoting the scientific evidence.

4. Discuss with patients the scientific rationale of any treatment I am considering to address their problems and why I am suggesting a certain course of care.

5. Avoid suggesting a treatment approach to a patient without discussing the expected benefits, the possible adverse reactions and the options that are available either through my office or by referral to another clinician.

6. Determine the preferences of my patient for the different treatment options when the likely outcomes are similar and empower him or her with the knowledge to make an educated decision on his or her care.

7. When a treatment option is decided on, I attempt to closely monitor the patient's positive and negative response to the treatment and make adjustments to the type of care offered depending on the response.

This does not preclude my right to offer a treatment approach that is off-label and for which there is limited evidence of effectiveness. I could not practice as a neurologist without this ability. It has been estimated that between 50-80 per cent of all treatments prescribed by medical physicians and specialists are off-label or have limited scientific support. There are many times when patients have tried all available evidence-based treatments without success and are requesting and are willing to try treatments based solely on my experience and recommendation. In this situation, however, I am very careful to tell the patient that there is no scientific support for the treatment we are considering, that no guarantees can be made for its success and that there are potential complications that may not be known. I am then willing to consider this approach for a limited period of time and discontinue the treatment if there is no positive response or a negative response becomes evident. I also avoid offering a treatment approach for which there is evidence that it is unlikely to be helpful, if the expense is too high to warrant the trial of what is essentially an experimental procedure or where the complication rate is known to be significant.

The chiropractic profession is to be congratulated on formulating this Evidence Report. It should be of considerable help to practicing chiropractors who are trying to practice according to the best scientific evidence, to patients who are seeking care and trying to decide whether chiropractic is a reasonable option, to other physicians who wish to refer patients to or work closely with chiropractors and to policy makers who have to decide what treatments should be paid for. The primary weakness of studies such as this is that they reflect the evidence at the time of publication. Evidence on manipulation and other treatment approaches offered by chiropractors is advancing every year and I hope that we will see routine updates of this document so that we, as physicians and the chiropractors we work with, can provide better care to our patients.

### Commentary on effectiveness of manual therapies by Professor Martin Underwood

The effectiveness, or otherwise, of manual therapies is the subject of considerable debate. It sometimes appears that this, occasionally heated, debate is fuelled more by the prior beliefs of the protagonists than by a rational examination of the evidence. This evidence report brings together a summary of all the randomised controlled trial evidence and guideline recommendations for manual therapies. Importantly, this has focussed on the treatments offered, rather than the professional background of the therapist. Many, but not all, of these treatments may be delivered by therapists with conventional biomedical training, such as physiotherapists or by complementary practitioners such as osteopaths or chiropractors. Understanding the evidence for, or against, the use of manual therapy for different disorders is far too important to allow it to be used in a debate of the integrity of particular professional groups. Manual therapies are characterised by the use of the therapist's hands; thus they include massage, joint mobilization within the normal range of movement, or manipulation taking a joint beyond its normal range of movement. Any consideration of the effectiveness of manual therapies also needs to recognise that non-specific factors such as the interaction between the therapist and the patient may have a therapeutic effect, in addition to any specific effect resulting from the manual treatment itself. From an academic perspective, it is of considerable interest to be able to quantify the specific and non-specific effects of any particular treatment. From a patient perspective, however, knowing whether an overall package of care, which includes manual therapy, has shown to be effective, is probably of greater relevance.

Any new drug treatments need to provide evidence of effectiveness prior to being marketed. In contrast new manual therapy approaches, some with a very poor theoretical underpinning, can be introduced and achieve popularity without any evidence of effectiveness being available. Few, if any, trials of manual therapy have been designed to show that an established treatment is ineffective. Many negative trials are too small to have been certain that an important therapeutic effect has not been overlooked. Thus, it is important when reading this report to remember that absence of evidence of effectiveness is not the same as evidence of absence of effectiveness.

Minor, self limiting, adverse effects such as muscle soreness following manual therapy are common. Serious adverse events are rare. Good data on their frequency are not available - these need to come from observational studies rather than randomised controlled trials. Manual therapists do need to counsel their patients about the risk of both minor and serious adverse events. For manipulation of the lumbar spine in an otherwise fit young adult with non-specific low back pain the risk of a serious adverse event is probably not of great concern. On the other hand, manipulation of the cervical spine of someone who has recently sustained a significant whiplash injury should probably be avoided. Additionally, there is the hazard that consulting a manual therapist, for a treatment that has not been shown to be effective, may stop the patient seeking appropriate medical treatment. This may not be so important for a child previously diagnosed with infantile colic, a minor self-limiting disorder, for which medical treatment is largely ineffective. On the other hand choosing manual therapy for a potentially fatal condition, such as asthma, in preference to established drug treatments would be unwise.

Notwithstanding these provisos, the key messages from this report are that:

• there is evidence to support the use of manual therapies for a range of, primarily musculoskeletal, disorders for which it is biologically plausible that they might have a specific effect

• there is not evidence for their use for a range of other disorders for which a biologically plausible mechanism for a specific effect is unclear

Thus, for example, the evidence supports use of manual therapy for non-specific low back pain and it does not support its use for enuresis or otitis media. Wherever possible we should use treatments of proven effectiveness. This dictum applies equally to the medical profession and to manual therapists. If a manual therapist is asked to treat a patient with a disorder for which they do not have a proven treatment approach they should first consider if a non-manual treatment would be more appropriate. If they do proceed to treat the patient, they need to explain to the patient the strength of the available evidence for effectiveness and what is known about potential adverse events. The vast majority of osteopaths and chiropractors in the UK are in private practice. This could lead to a concern that unproven treatments are being inappropriately offered for short-term commercial gain. Similar concerns might be raised for my medical colleagues who work in private practice. Such unprofessional behaviour should be avoided by all professions.

For some non-musculoskeletal disorders for which manual treatment has achieved popularity, without evidence of effectiveness being available there is a need for new trials to produce definitive evidence of effectiveness/ineffectiveness of manual therapy. In the meantime, this excellent report gives clear guidance on the disorders for which the use of manual therapy is supported by objective evidence of effectiveness. I recommend this report as essential reading for all manual therapists before considering which treatments they should offer, and the information they give, to their patients.

## Competing interests

SH has served or continues to serve on a number of Guideline panels that have dealt with some of the topics included in this study. These committees have been established by the North American Spine Society, the United States (US) Government Agency for Health Care Policy and Research (AHCPR), the Bone and Joint Decade 20000-2010 Task Force on Neck Pain and Its Associated Disorders (NPTF), Guidelines for Chiropractic Quality Assurance and Practice Parameters, the American Academy of Occupational and Environmental Medicine and the California Department of Industrial Relations. He is not currently the recipient of any research grant or support funding. He does serve as a consultant to Palladian Health. He is currently president of World Spine Care, a charitable non-profit organization established with the goal of helping people in underserved regions of the world who suffer from spinal disorders.

MU was one of the principal investigators on the UK BEAM trial of manipulation and exercise for low back pain which found a package of manual therapy to be effective for low back pain; he was chair of the National Institute of Health and Clinical Evidence (NICE) guideline development group that developed guidelines on the early management of persistent low back pain that recommended that manual therapy as a treatment option; he is a co-applicant on two current research projects into the incidence of adverse events following manual therapy funded by the National Council for Osteopathic Research

## Authors' contributions

Both authors contributed equally to this manuscript and provided their commentaries independently. The journal editors combined their commentaries into this single paper.
